# Exogenous Cytokinin Induces Callus and Protocorm-Like-Bodies Formation in *In Vitro* Root Tips of *Vanilla planifolia* Andrews

**DOI:** 10.21315/tlsr2024.35.1.13

**Published:** 2024-03-30

**Authors:** Li Chin Chai, Peter G. Alderson, Chiew Foan Chin

**Affiliations:** 1 School of Biosciences, Faculty of Science, The University of Nottingham Malaysia Campus, Jalan Broga, 43500 Semenyih, Selangor, Malaysia; 2 School of Biosciences, The University of Nottingham Sutton Bonington Campus, Loughborough, Leicestershire LE12 5RD, United Kingdom

**Keywords:** *Vanilla planifolia* Andrews, Root Tips, Callus, PLB, *In vitro*, Cytokinin, *Vanilla planifolia* Andrews, Hujung Akar, Kalus, PLB, *In vitro*, Sitokinin

## Abstract

Vanilla is a popular flavouring essence derived from the pods of vanilla orchid plants. Due to the high demand for vanilla flavour, high yielding vanilla plantlets are necessary for establishing vanilla plantations. Clonal micropropagation is a viable technique for the mass production of high yielding vanilla plantlets. This study reports an efficient regeneration protocol by using cytokinin as the sole plant growth regulator to regenerate plantlets from the root tips of a commercial vanilla orchid species, *Vanilla planifolia*. Most studies to date have reported using seeds and nodes as starting explants for *in vitro* micropropagation of vanilla orchids. So far, regeneration from roots has not been very successful. Previous studies favoured the use of auxins only or high auxin to cytokinin ratios to induce callus, and sole cytokinins were used for direct shoot regeneration. However, it was sporadically observed in plantlets regeneration of *V. planifolia* that multiple shoots were regenerated from the tips of intact aerial roots submerged in media. This study therefore investigated the regeneration of excised vanilla root tips through the application of most commonly used auxins (1-naphthaleneacetic acid and 2,4-dichlorophenoxyacetic acid) and cytokinins (6-benzylaminopurine and thidiazuron). High auxin presence is known to promote callusing in *in vitro* plants. However, in this study, auxin treatment inhibits callusing in root tips. While cytokinin treatments, even at low levels, has promoted high rate of callusing. These callus cells regenerate into protocorm-like-body (PLB) shoots when cytokinin levels are increased to 0.5 mg/mL 6-benzylaminopurine (BAP) under light conditions. The findings of the study have the potential of providing large quantity of high yielding vanilla plantlets through clonal micropropagation.

## Abstract (ms)

Vanila ialah esen perasa popular yang diperoleh daripada buah pokok orkid vanila. Disebabkan permintaan yang tinggi untuk perisa vanila, penghasilan anak benih vanila yang banyak diperlukan untuk membuka ladang vanila. Pembiakan mikro klonal adalah teknik yang berdaya maju untuk pengeluaran besar-besaran plantlet vanila. Kajian ini melaporkan protokol penjanaan semula yang cekap dengan menggunakan sitokinin sebagai pengawal selia pertumbuhan tumbuhan tunggal untuk menjana semula tumbuhan daripada hujung akar spesies orkid vanila komersial, *Vanilla planifolia*. Sehingga kini, kebanyakan kajian telah menggunakan benih dan nod sebagai eksplan permulaan untuk pembiakan in vitro orkid vanila. Setakat ini, penjanaan semula dari akar tidak begitu berjaya. Kajian terdahulu mengutamakan penggunaan auksin sahaja atau nisbah auksin tinggi kepada sitokinin untuk mendorong kalus, dan sitokinin tunggal digunakan untuk penjanaan semula pucuk langsung. Walau bagaimanapun, secara sporadis diperhatikan dalam penjanaan semula tumbuhan *V. planifolia* bahawa beberapa pucuk telah dijana semula daripada hujung akar udara utuh yang tenggelam dalam media. Oleh itu, kajian ini menyiasat penjanaan semula hujung akar vanila yang dipotong melalui penggunaan auksin yang paling biasa digunakan (asid 1-naftalenaasetik dan asid 2,4-diklorofenoksiasetik) dan sitokinin (6-benzylaminopurine dan thidiazuron). Kehadiran auksin yang tinggi diketahui menggalakkan kalus dalam tumbuhan *in vitro*. Walau bagaimanapun, dalam kajian ini, rawatan auksin menghalang kapalan di hujung akar. Walaupun rawatan sitokinin, pada tahap yang rendah, telah menggalakkan kadar kalus yang tinggi. Sel kalus ini menjana semula menjadi pucuk jasad berbentuk protokom (PLB) apabila paras sitokinin dinaikkan kepada 0.5 mg/mL 6-benzylaminopurine (BAP) di bawah keadaan cahaya. Dapatan kajian mempunyai potensi untuk membekalkan tumbuhan vanila hasil tinggi dalam kuantiti yang banyak melalui pembiakan mikro klon.

**Highlights**

- Root tips of *Vanilla planifolia* have high pluripotency.
- The use of 6-benzylaminopurine (BAP) or Thidiazuron (TDZ) alone at low concentrations can induce proliferation of callus leading to formation of shoots and plantlets.
- The protocol is a potential tool for mass propagation of *Vanilla planifolia*.

## INTRODUCTION

Vanilla flavour produced from the seed pods of edible orchid *Vanilla planifolia*, is the second most expensive spice in the world, after saffron (*Crocus sativus*) (Mani *et al*. 2017; Gaikwad *et al*. 2023). It is widely used as the most popular flavouring agent for numerous sweetened foods worldwide (Spence 2022) as well as the most promising sweet enhancing aroma (Bertelsen *et al*. 2020) and sugar-reduction flavour (Oliveria *et al*. 2021). In addition, it is also broadly used in perfumery, cosmetic and medicinal industries (Busconi *et al*. 2017; Retheesh & Bhat 2011). Its major compound, vanillin, has been found to have wound healing (Sinsuebpol *et al*. 2023), vasodilatory (Choi *et al*. 2023), neurodegenerative/anti-neuroinflammatory/anti-neurotoxicity (Iannuzzi *et al*. 2023; Zhong *et al*. 2019; Kim 2019), anti-cancer (Li *et al*. 2021), antidepressant and appetite enhancing (Chuang *et al*. 2020; Ogawa *et al*. 2018), antibacterial (Mok *et al*. 2020), and kidney injury (diabetic nephropathy) protection (Tong *et al*. 2019) effects. This makes *V. planifolia* the most profitable orchid worldwide (Azofeifa-Bolaños *et al*. 2017). Currently, the high demand for natural vanillin has led to the need for expansion in areas for planting high quality vanilla plants, which in turn led to the high demand for this plant as a planting stock.

Traditionally, *V. planifolia* is propagated by stem-cuttings. However, this method is labour intensive, time consuming (Kalimuthu *et al*. 2006; Janarthanam & Seshadri 2008) and not economical, as the collection of the stem cuttings from mother plants may eventually cause retardation to their growth and reduce their yield potential (Davidonis & Knorr 1991; Geetha & Shetty 2000; Kalimuthu *et al*. 2006). Traditional seed germination method was also not viable because the germination rate of vanilla seeds has been low (Tan *et al*. 2011a). Like other orchids, the seeds of *Vanilla planifolia* Andrews rarely germinate because of the presence of germination inhibitors (Dequaire 1976) and its dependency on the presence of mycorrhizal fungi (Arditti & Ghani 2000). Therefore, plant tissue culture offers a viable alternative route for the mass production of vanilla orchid plantlets.

Both callus and protocorm-like bodies (PLBs) play vital roles in orchid micropropagation. Callus refers to the undifferentiated and unorganised cell mass resembling wound healing plant tissue, with implications for its potential to undergo cell proliferation and differentiation (Fehér *et al*. 2019). On the other hand, PLBs are differentiated cells that structurally resemble somatic embryos derived from seeds (protocorms) but induced from vegetative explants and/or calluses (Cardoso *et al*. 2020).

PLBs are the ideal explants for *in vitro* propagation due to their ability to generate secondary PLBs and differentiate into complete plants (Guo *et al*. 2021). They are widely used in the orchid floriculture industry for clonal propagation through cuttings (Yam & Arditti 2009; Fang *et al*. 2016). PLBs offer an efficient and preferred method for rapid multiplication, easily achieved through subculture, resulting in a large number of PLBs obtained in a short period. Their well-differentiated tissues facilitate rapid regeneration into complete plantlets, significantly reducing time and resources require for micropropagation (Mohanty *et al*. 2012). Additionally, PLBs can be cryopreserved for long-term storage of valuable genetic resources, which support conservation efforts (Das *et al*. 2021). Both callus and PLBs are commonly utilised in transgenic regeneration (Shrestha *et al*. 2010; Cardoso *et al*. 2020).

**Figure 1:**
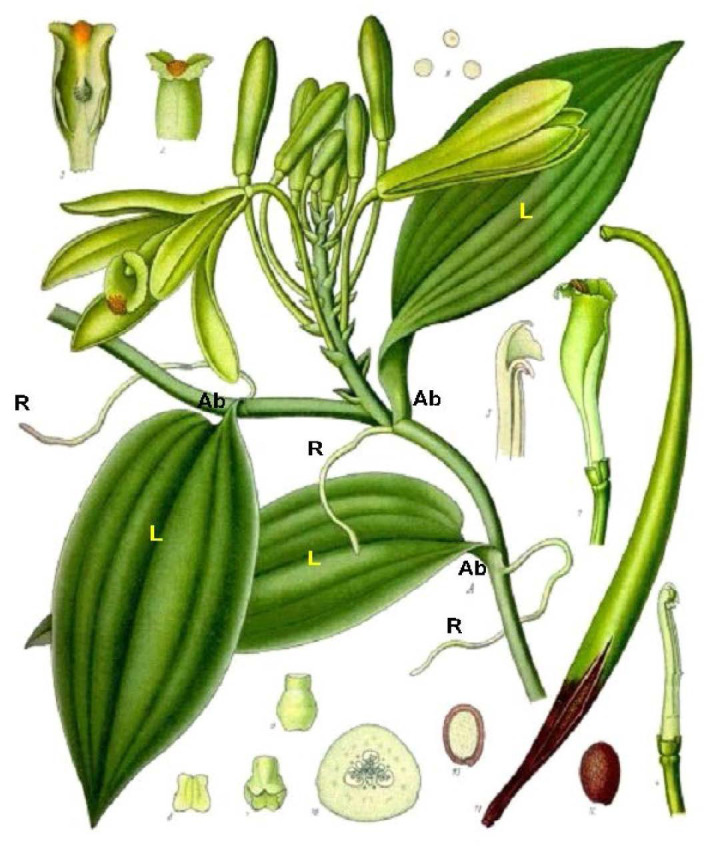
Schematic diagram showing *V. planifolia* plant forms: axillary buds, leaves and aerial roots are abundant at each nodes of the plant. R = aerial roots; Ab = axillary buds (wrapped in between leave bases and nodes); L = leaves. (Source: Arditti 2019).

In addition, root tips culture could be a useful alternative propagation method to nodal culture, with the major advantage of propagating the plants without sacrificing the monopodial mother plant, as opposed to the current nodal regeneration method (Philip & Nainar 1986). Aerial root tips are explants of plentiful supply, with at least one root tip present on each node of a healthy vanilla plant (Fig. 1) (Arditti 2009). The aerial roots of vanilla, like other epiphytic orchids, are rather unique. The entire length of the root, except the tip, is covered by dead and spongy-like velamentous tissues which function for water and nutrient uptake, as well as for protection against UV-B damages. Underneath the surface of the aerial roots lies the cortex region containing chloroplasts (Chomicki *et al*. 2014; Zotz & Winkler 2013). The orchid roots may form as much as half the total plant mass, which function in absorption of mineral, carbon fixation, water conservation and anchorage (Zhang *et al*. 1995).

*In vitro* regeneration of *V. planifolia* has been developed through shoot tip and axillary bud (Geetha & Shetty 2000; George & Ravishanka 1997; Giridhar & Ravishankar 2004; Kalimuthu *et al*. 2006; Gopi *et al*. 2006; Lee-Espinosa *et al*. 2008; Tan *et al*. 2011b; Inderiati *et al*. 2019; Latif *et al*. 2019; Erawati *et al*. 2020; Manokari *et al*. 2021; Thye *et al*. 2023), root apex (Philip & Nainar 1986), leaf (Gu *et al*. 1987; Janarthanam & Seshadri 2008; Tan *et al*. 2011a) and seed-derived PLBs (Malabadi & Nataraja 2007; Retheesh & Bhat 2011; Yeh *et al*. 2021; Šoch *et al*. 2023). Micropropagation of *V. planifolia* using nodes as explants has been reported to have successfully produced approximately 100,000 to 150,000 plantlets per explants from each mother plant within 12 months (Lee-Espinosa *et al*. 2008). However, the availability of nodes is limited compared to roots which are abundantly available at almost every single node of a vanilla plant (Fig. 1). Up until today, only a few studies have been reported on the micropropagation of *V. planifolia* via roots. Earlier study revealed poor callusing through using roots as explants (Philip & Nainar 1986). In more recent years, Tan *et al*. (2011a) reported only 35% and 10% of callus from nodal and leaf explants respectively when cultured on MS medium containing 2.0 mg/L 1-naphthaleneacetic acid (NAA) and 1.0 mg/L 6-benzylaminopurine (BAP). In this study, callus and protocorm-like bodies (PLBs) formation were sporadically observed in the aerial root tips of *V. planifolia* submerged in Murashige and Skoog (MS) medium supplemented with 1.0 mg/L BAP. These calli and PLBs subsequently regenerated into multiple shoots with the maternal roots still intact and were able to grow into independent plantlets in the same medium. Thus, the objective of this study is to investigate this *in vitro* growth and development of *V. planifolia* by applying different hormonal treatments in excised root tips, using nodal cultures as a reference experiment.

## MATERIALS AND METHODS

### Source of Plant Materials

Explants used for tissue culture were obtained from the *V. planifolia* plants grown in the shade house of University of Nottingham Malaysia Campus. The original source of *V. planifolia* cuttings were purchased from the nursery of Kuasa Sekata Sdn Bhd, Selangor, Malaysia. Juvenile nodal segments (1.5 to 2.0 cm) of *V. planifolia* were surface disinfected according to the protocol by Tan *et al*. (2010a) with slight modification. The cleaned explants were cultured onto full-strength Murashige and Skoog (MS) solid medium containing 1.0 mg/L BAP for one month.

### Media and Culture Conditions

Full-strength and half-strength Murashige and Skoog (MS) media were prepared using 4.43 g/L and 2.215 g/L of MS-basal salt (*Phyto*technology Laboratory^®^, USA), respectively, with the addition of 3.0% (w/v) sucrose (Fisher Scientific, USA) and 3.5% (w/v) PhytagelTM (Sigma-Aldrich, USA). For liquid medium, the addition of Phytagel^™^ was omitted. The MS media were adjusted to pH 5.8 ± 0.2 with 1 M NaOH or 1 M HCl before autoclaved for 15 min at 121°C and 15 psi pressure. All plant growth regulator stock solutions were prepared at a concentration of 1.0 mg/mL, filtered through 0.2 μm filter (Minisart^®^, Sartorius) in the laminar air flow cabinet, and stored in the 4°C fridge for up to six months. Light-sensitive hormones, such as Thidiazuron (TDZ), were stored in the dark at 4°C. All cultures were incubated at 25 ± 2°C under 16:8 light-dark cycle with a light intensity of 40 μmol m^−2^ s^−1^ provided by cool white fluorescent lights. The temperature and relative humidity (RH) of culture rooms were monitored every 25 min with a HOBO Wireless Data Logger System (OnSet, USA). Callus induction was carried out under both light and dark conditions, while shoot regeneration was carried out under light condition.

### Establishment of aseptic cultures through surface sterilisation

Juvenile nodal segments (1.5 to 2.0 cm) of *V. planifolia* were surface disinfected according to the protocol by Tan *et al*. (2011a) with slight modification. Briefly, the nodal segments were first cleaned thoroughly by gently brushing under running tap water for 10 min and washed in liquid detergent containing one to two drops of Tween 20 in 1.0 L distilled water, followed by three rinses with sterile distilled water. Further surface disinfection was carried out by submerging and shaking the explants in 0.2% (v/v) carbendazim (Mei Zim, Malaysia) for 20 min, rinsing thrice with sterile distilled water, immersing and shaking in 70% (v/v) ethanol for 2 min followed by washing thrice with sterile distilled water. The cleaned explants were then surface sterilised in 20% (v/v) Clorox^®^ (commercial bleach containing 5.25% w/v sodium hypochlorite) with the addition of a few drops of Tween 20 for 20 min with constant shaking inside a laminar air flow cabinet and finally rinsed thrice with sterile distilled water before cultured on full-strength MS solid medium containing 1.0 mg/L BAP for one month. After one month, nodes were cultured in full-strength MS liquid medium without plant growth regulators for a week. Shoots regenerated from clean nodal explants free of microbial contamination were used for subsequent callus and shoot regeneration experiments. This surface sterilisation protocol was further optimised for various carbendazim concentrations used (0.2%, 0.4%, 0.6%, 0.8% and 1.0%) and incubation durations (20 min, 60 min, 120 min and 24 h). The aseptic cultures established in this protocol were used as explants for further plant growth regulators optimisations. Data of the percentages of contamination and phytotoxicity were recorded after one month from culture initiation.

### Vanilla Nodal, Leaf and Root Tips Culture

Juvenile vanilla nodal, leaf, root tip explants from three-month-old *in vitro* vanilla plantlets were cultured on half-strength MS media supplemented with 1.0 g/L myoinositol, 1.0 mg/L thiamine-HCl in various concentrations of NAA, 2,4-D and BAP combinations for callus induction or shoot initiation (see Tables 2, 3 and 4). For nodal segments culture (NT), nodal segments containing one intact axillary bud, with its attached leaf removed, were cultured. For root tips regeneration (RT), 0.5 ± 0.1 cm of aerial root tips were cultured. For leaf apices and bases (LT), 0.5–1.0 cm in diameter from leaf tip and leaf bases respectively were used and the explants were placed with the adaxial surface in contact with the solid culture medium (Arditti 2006). The percentage of explants forming callus/shoot, the average size of callus and number of shoots formed were recorded after three months of culture. For each experimental treatment, three replicates each consisting of five units were carried out. The percentage of callus and shoot induced, callus sizes and weights, and number of shoots formed per explant were measured after 90 days of culture.

### Shoot Proliferation and Maintenance and Rooting

Successfully generated calli (30 ± 5 mg) were transferred to full-strength MS medium supplemented with 1.0 mg/L BAP (nodal culture) and 0.5 mg/L BAP (root tip culture) for shoot initiation and elongation under light condition. Elongated shoots were maintained on the same medium every four weeks for three months. The percentage of callus cultures forming shoots, number of shoots formed per callus and average shoot length were recorded after six months of culture. Shoots of 2–4 cm in height were transferred for root induction on full-strength MS basal medium supplemented with NAA (Tan *et al*. 2011a).

### Morphological Observation and Histological Analysis

Morphogenesis observation of callus (derived from nodes and roots), single or multiple shoot regeneration, and any phenotypic abnormalities from the *in vitro* regenerants, were observed macroscopically, and microscopically using dissecting microscope, Nikon MZ1500 (Nikon, USA). For histological analysis, plant cells were fixed for 24 h to 48 h at room temperature in a Glutaldehyde-Paraformaldehyde-Caffeine (GPC) (Sigma-Aldrich, USA) fixative solution, then dehydrated in series of ascending ethanol series at 30–100% (v/v) and infiltrated with basic resin (Leica Historesin Embedding Kit, Germany) for 1–2 weeks. Subsequently, the plant cells were embedded and polymerised in a mould overnight. Serial sectioning was then performed at 4 μm using Leica RM2165 rotary microtome (Leica Microsystems, Germany). The samples were stained with toluidine blue staining reagent (Sigma-Aldrich, USA) for checking. Finally, the slides were stained with periodic acid, Schiff’s reagents and Naphthol Blue Black reagent (Sigma-Aldrich, USA) and mounted onto Cytoseal^™^ 60 mounting medium. The sample slides were viewed under light microscope, Nikon Eclipse 80i (Nikon, USA) and all microscopic photos were captured with camera through the NIS-Element 3.0 software (Nikon, USA).

### Statistical Analysis

The experiments were conducted in a completely randomised block (CRB) design with three replicates of five culture units for each treatment. Data in percentage were transformed (arcsine) using logistic regression analysis, at the assumptions of generalised linear models. All data were also subjected to one-way analysis of variance (ANOVA) using GenStat 18th Edition software. Duncan’s multiple range tests (DMRT) with significant difference level at *p* ≤ 0.05 was then used to analyse the differences between the treatments.

## RESULTS

### Improvement on The Method of Establishment of Vanilla Aseptic Cultures

Using the surface sterilisation method developed by Tan *et al*. (2011a), the vanilla nodal cultures had an average of 80%–90% bacterial contaminations and 50%–60% fungal contaminations. Bacterial contaminations were mostly observed within two weeks, but fungal contamination was detected even after one month. Therefore, observations at post-surface sterilisation were carried out for at least a month to ensure the cultures were truly contamination-free. Increased carbendazim concentration to 0.4% has successfully controlled the fungal contamination, while further increase in carbendazim concentration at 0.6% and above caused phytotoxicity to the explants (Table 1). Therefore 0.4% carbendazim was used for subsequent optimisation of submergence duration. Table 1 shows that bacterial contamination (up to 90%) were only controlled when the duration of carbendazim treatment was prolonged to 60 min. Overall, 0.4% carbendazim for 60 min was the optimal protocol for reducing fungal and bacterial contamination in aseptic culture of vanilla.

### Nodal Culture

**Figure 2:**
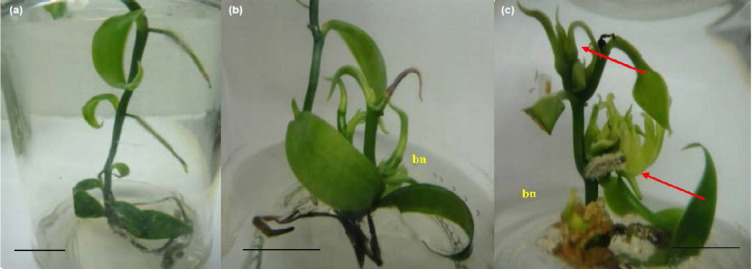
Direct single, multiple and abnormal shoot regenerations of vanilla nodal cultures. Nodal segments when treated with (a) 0.0 (b) 1.0 and (c) 5.0 mg/L BAP for 4 months; bn: basal node; red arrow indicates the germination of axillary buds at multiple aerial nodes other than the basal node. Bar = 1.0 cm.

Direct and indirect shoot regenerations were observed (Table 2). Nodal explants went through direct regeneration when treated with BAP alone. Without phytohormones (control treatment), vanilla nodes produced an average of only one shoot per node (Fig. 2a, Table 2). Increasing BAP concentrations promoted multiple shoot responses of vanilla. Across the BAP concentrations, optimal shoot response was achieved with the treatment of 1.0 mg/L BAP (Fig. 2b), at an average of 8.0 shoots per node after 90 days of culture (Table 2). Higher BAP concentrations caused abnormality in vanilla nodes. At normal conditions, shoot multiplications occurred only at the axillary buds of basal nodes (bn). However, at 5.0 mg/L of BAP, shoots exhibited abnormal phenotypes, all axillary buds on vanilla aerial nodes germinated and formed shoot primordia (Fig. 2c). These eventually failed to develop into proper shoots that could be regenerated.

The addition of NAA to BAP induced callusing in vanilla. The optimal treatment among NAA treatments was 2.5 mg/L with 1.0 mg/L BAP, with the highest callusing rate (66.67%) and the highest number of shoots formed after 6 months (an average of 10.5 shoots per callus) (Table 2). The callusing rate of vanilla increased with NAA concentrations, when treated in combination with 2.5 mg/L BAP. At lower levels of NAA (0.5 mg/L), direct multiple shoot multiplications were promoted mostly without going through the callus phase. At the median levels of NAA (1.0 mg/L), green calluses were formed. While at the higher levels of NAA (2.5 mg/L), calluses formed were yellowish and compact (data not shown).

Varied BAP levels treated in combination with 2.5 mg/L NAA changed the callus morphology of vanilla. At lower levels of BAP concentration (0.5 mg/L), calluses were less de-differentiated and darker green in colour. At median level of BAP concentration (1.0 mg/L), calluses were more embryogenic-looking and light green in colour. While at higher level of BAP concentration (2.5 mg/L), calluses appeared to be more de-differentiated, compact and yellow in colour.

The addition of 2,4-D also induced callusing in vanilla. Callus formation is the highest when nodes were treated with 0.5 mg/L or 1.0 mg/L of 2,4-D in combination with 0.5 mg/L BAP (Table 2). However, 2,4-D concentrations at 1.0 mg/L and above, caused explants browning over time, showing phytotoxicity effect to the vanilla plants. Even though calluses were formed, the original explants showed stress symptoms, with its internode and leaves wilted and turned brown eventually.

### Root Tips Culture (RT)

**Figure 3:**
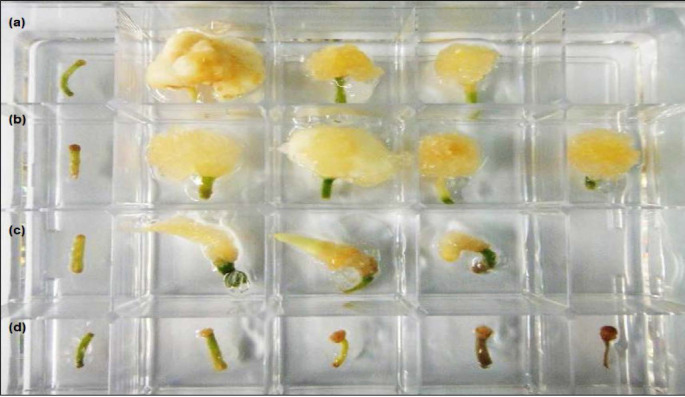
Response of in vitro root tips culture to different hormones. Concentrations from low to high, from left to right: (a) BAP (0, 0.5, 1.0, 2.0 mg/L), (b) TDZ (0, 0.1, 0.25, 0.5, 1.0 mg/L), (c) NAA (0, 0.5, 1.0, 2.0 mg/L), (d) 2,4-D (0, 0.1, 0.5, 1.0, 2.0 mg/L) after 90 days.

*In vitro* culture of vanilla root tips formed callus under the influence of cytokinins, instead of auxins. In the control treatment (without the addition of any phytohormones), callusing rate was zero but root elongated. It was shown that high callusing rates (86.67 to 100.00%) were achieved with cytokinin treatments alone (TDZ or BAP) of root tips (Table 3, Fig. 3). While under auxin treatments alone (NAA or 2,4-D), root tips neither elongated nor callused. When cytokinin was interacted with auxin treatments, the combination of auxins and cytokinins produced callus ranging from 20.00 to 100.00% (Table 4). However, the average size of callus formed in auxin-cytokinin combinational treatments were much smaller than the cytokinin-only treatments after 90 days (Tables 3 and 4).

Surprisingly, callus cultured under light conditions did not exhibit responsiveness to callusing (data not presented). This observation contradicts the initial observation in *in vitro* plantlets, where occasional callusing and PLB formation was observed when plantlets were cultured in MS supplemented with 1.0 mg/ML BAP under light conditions. This discrepancy is likely attributed to the endogenous hormone levels, as light also plays a crucial role in callus induction in roots. Our results demonstrate that callus induction under dark conditions consistently and significantly promotes callus growth.

In terms of shoot induction and proliferation, the results show that for the callus cells to regenerate into shoots, cytokinin levels need to be at least 0.5 mg/mL (Table 2). MS media (hormone-free) did not induce any shoot differentiation in root-tip derived callus cells. Callus cells regenerated into PLBs only in MS supplemented with 0.5 mg/mL. This highlighted the role of higher concentrations of cytokinin in addition to the presence of light for shoot regeneration of *V. planifolia* callus from root tips.

### Histological Analysis of Root Tips Structure

**Figure 4:**
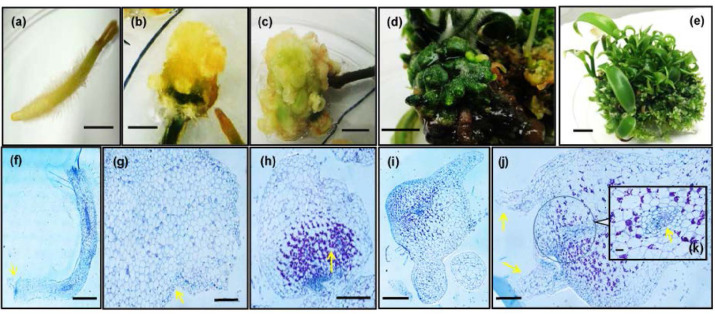
**(a–e)**. The progressive development of root-derived callus to PLB and multiple shoots mass of root tips of *V. planifolia*: (a) Elongating root tips prior to hormone treatments; (b) 90-days-old and (c) 150-days-old root-derived callus; (d) Differentiating shoot primodia and (e) multiple shoots derived from root callus culture. Bar = 0.5 cm. (f–j) Light micrograph showing the sequence of development of callus from cross section of vanilla root tip: (f) Root tip (two weeks after incubation in callus inducing medium), arrow showing root tip, bar = 2,500 μm; (g) Heart shape embryo-like structure, arrow showing tunica corpus organisation, bar = 500 μm; (h) Meristematic cells with changes in starch accummulations (dark purple stained granules, as indicated by arrows), bars = 500 μm; (i) Meristematic structures forming shoot apex, bar = 500 μm; (j) Shoot apex with leaf primodia and development of vascular tissues in protocorm-like structure, arrow shows distinct leaf primordia formation, bar = 500 μm; (k) vascular bundles formation in PLB structure, bar = 50 μm.

Histological analysis was carried out using root tips at different developmental stages (Fig. 4): elongating root tips prior to hormone treatments (Fig. 4a), root tips after two weeks cultured in 0.5 mg/L BAP, initial stage of root tips-derived calluses and 3-months-old root tips-derived calluses in shoot regeneration media. At macroscopic level, the calluses formed from root tips were mostly compact, irregular and light yellow in colour (Fig. 4b), turning from light green (Fig. 4c) to dark green (Fig. 4d) in the progress of differentiating into PLB masses (Fig. 4e). Under histological analysis, it was observed that after two weeks of incubation in callus-inducing medium, root caps in root tips started to disintegrate (Fig. 4f). At the initial callus stage, cells were globular and highly vacuolar. Heart-shaped embryo-like structures with meristematic globular cells (Fig. 4g) started to form in 3-months-old callus after incubated in shoot regeneration media. Polysaccharides accumulations (dark purple stained granules in Fig. 4h) were observed as the callus cells began to regenerate and shoot primordial (Fig. 4i–4j) were formed with vascular bundles formation (Fig. 4j) in the more differentiated protocorm-like structures.

## DISCUSSION

### Fungal and Endophytic Bacterial Contaminations as Challenges in Establishment of Vanilla Aseptic Cultures

Diseases are one of the main factors that damage and reduce vanilla production and productivity. The conditions of temperature and humidity under which vanilla grows tend to favour the development of pathogens, mainly fungi (Havkin-Frenkel & Belanger 2011). Vanilla plantations were often devastated by root and stem rot (*Fusarium oxysporum* f. sp. *vanillae)*, black rot (*Phytophtora* sp.) and anthracnose (*Colletotrichum* sp.), sclerotium rot (*Sclerotium rolfsii*) and rust (Uromyces sp., Puccinia sp.) (Havkin-Frenkel & Belanger 2011; Carbajal-Valenzuela *et al*. 2021). These airborne fungi could be trapped easily in the spaces between the axillary bud and leaves of vanilla nodes, which were not readily removable and could be easily carried into *in vitro* culture systems. This could be problematic as some fungus could even appear after one month of culture. Fungal contamination was the first major problem faced in this study during the establishment of sterile culture from the shade house grown vanilla, and was successfully eliminated with increased carbendazim concentration from 0.2% to 0.4% (Table 1). Carbendazim is a broad-spectrum benzimidazole fungicide. The breakdown product of thiophanate methyl and benomyl have strong inhibition on *Fusarium* sp., which was the main fungal pathogen in vanilla culture. Carbendazim is also effective against other fungal pathogens such as *Botrytis cinerea* (Bi *et al*. 2009; Debergh *et al*. 1993).

The second major issue faced in this study was the high bacterial contamination (up to 90%). These bacteria contamination did not all appear immediately after surface sterilisation. Some could appear after two weeks to a month, and thus could potentially be endophytic bacteria. Vanilla, like other orchids, has systemic endophytes which have both phyto-beneficial and biocontrol capacities (Lalanne-Tisné *et al*. 2023). They could help in the plant’s growth and development especially during seedlings development (Faria *et al*. 2013; White *et al*. 2014) and also act as potential biocontrol agent for fungal diseases (Karol *et al*. 2015; Lalanne-Tisné *et al*. 2023). These bacteria were harmless inside the plants, even at the initial stages of *in vitro* culture system. However, as suggested by Herman (1989), the usually non-pathogenic or even the growth-promoting bacteria can become detrimental under the special growth conditions of *in vitro* culture, which was termed as “vitropaths”. We observed that eventhough some endophytic bacteria occurred in vanilla cultures did not affect plant growth, occasionally some bacteria tend to outgrow the plant, and eventually killed the plant and colonised the entire medium, thus affecting other downstream tissue culture processes. Prolonged exposure to carbendazim from 20 min to an hour has successfully reduced the endophytic bacteria contamination by down to 10% (Table 1). This study showed that although carbendazim is a fungicide, it also had bactericidal effects as reported by Garcia *et al*. (2002), Wang, Song, *et al*. (2009; 2011), and Wang, Huang, *et al*. (2009).

Besides having both fungicidal and bactericidal effects, carbendazim was also known to have enhancing effect on plant growth when used in low concentrations. Tripathi and Ram (1982) were the first to report enhancing effect of carbendazim on callus growth, root induction and shoot differentiations of *Daucus carota* culture. Furthermore, Shields *et al*. (1984) reported that carbendazim was not phytotoxic for callus and root cultures of *Nicotiana tabacum* at low dosages. Debergh *et al*. (1993) stated a cytokinin-like activity of carbendazim, with enhanced shoot height and increased number of shoots in *Cordyline terminalis* and showed no phytotoxicity effect of up to 160 μg/mL. We have observed that a low dosage (0.4% and below) and overnight incubation with carbendazim was harmless to vanilla cultures.

### Initiation of Shoots or Callus from Nodal Explants Was Influenced by the PGR Combination Treatments

Nodal regeneration experiments were carried out as a reference experiment to the culture of other vegetative tissues. Both direct and indirect shoot regenerations were observed when vanilla nodes were under the treatments of different PGR combinations.

#### Direct shoot regeneration

In general, the nodal culture of vanilla was very responsive to direct regeneration (Table 2). The treatment of 1.0 mg/L BAP has been the most widely reported optimal treatment for direct regeneration of vanilla (Sreedhar *et al*. 2007; Tan *et al*. 2011a; Inderiati *et al*. 2019). Our results similarly found that 1.0 mg/L BAP gave the optimal number of shoots formation (an average of 8.0 shoots per node) among the BAP treatments (0 to 5.0 mg/L) tested.

#### Indirect shoot regeneration

Incorporation of 1.0 mg/L BAP and 1.0 mg/L 2,4-D in the growth media results in the formation of an average of 50.0% callus response and 8.5 shoots. At concentrations above 1.0 mg/L 2,4-D, browning of the original explants were observed indicating cultured tissues were under stress. This could be caused by the herbicidal properties of 2,4-D (Phua *et al*. 2016). The auxin 2,4-D is an effective plant cell regulator in inducing cell elongation and enlargement commonly used for callus induction at low concentrations. The combination of 2.5 mg/L NAA and 1.0 mg/L BAP was the optimal PGR combination for callus induction (66.67% callus response) and its subsequent shoot regeneration (an average shoots number of 10.5).

This study shows that among the different nodal protocols used, the performance of nodal culture was consistent with the results from the previous research work (Tan *et al*. 2011a; Janarthanam & Seshadri 2008). Indirect regeneration protocol was able to produce more shoots than the direct regeneration protocol. The treatments 1.0 mg/L BAP and 2.5 mg/L NAA in combination with 1.0 mg/L BAP were optimal for direct and indirect regeneration, respectively, for nodal culture.

### Initiation of Callus via Vanilla Root Tips: Roles of Auxin vs. Cytokinin

The current study shows that the root tips of vanilla responded to the application of exogenous PGR in a very different manner compared to the nodal explants of vanilla. In general, an intermediate ratio of auxin and cytokinin promotes callus induction, while the presence of high ratio of auxins was known to promote callusing in *in vitro* plants (Skoog & Miller 1957). In nodal cultures, the application of exogenous auxins promoted the formation of calluses while the application of exogenous cytokinins promoted multiple shoots formations (Table 2). On the contrary, the application of exogenous auxins in root tips inhibits the induction of callus while the application of exogenous cytokinins promoted callus formation (Table 3). Besides, callus formation in nodal explants was formed at higher rate in high auxin to cytokinin ratios (Table 2), which differed from the response of root tips culture which was formed at higher cytokinin to auxin ratios (Table 4).

The results of the experiment (RT-A, Table 3) showed that high callusing rate (86.67% to 100.00%) were achieved with cytokinin treatments alone (TDZ or BAP) on root tips. While under auxin treatments alone (NAA or 2,4-D), root tips are neither elongated nor callused. Indicating that cytokinin is required for callus formation in vanilla root tips. In the auxin-cytokinin combinational treatments, the average sizes of callus formed were much smaller than the cytokinin-only treatments after 90 days. This further suggests that in vanilla root tips, exogenous application of auxins (NAA or 2,4-D) exhibited inhibitory effects on callus. Therefore, elevated cytokinin levels are required to reach a balanced auxin-cytokinin ratio for forming callus.

### Aerial Roots, A Natural Auxin Sink

During *in vitro* culture, the ratio of auxin and cytokinin determines the morphogenetic pathway that the *in vitro* cultured tissue will follow: high and low ratios of cytokinin to auxin favoured shoot and root regeneration, respectively, whereas more balanced concentrations resulted in callus formation (Skoog & Miller 1957; Novak *et al*. 2014). While auxins generally promote callus formation in many plants, the response can vary depending on the specific plant species, tissue type, and developmental stage. Our observation that high auxin does not promote callusing in vanilla root tips, while cytokinin does, suggests that there might be specific factors or mechanisms at play in the root tissue of this plant species. One possible explanation would be due to the interplay between auxins and cytokinins due to the endogenous level of these hormones in root tips.

Vanilla aerial roots, like the aerial root tips of other Crassulacean acid metabolism (CAM) orchids, might contain high endogenous auxin, cytokinin or abscisic acid contents (Zhang *et al*. 1995), which function to allow for its elongation, formation of lateral roots, gravitropism and control of root apical meristems (Aloni *et al*. 2006). This hypothesis could be supported by a study conducted by Neelannavar (2006), where IAA was measured using ultraviolet (UV), thin layer chromatography (TLC), gas liquid chromatography (GLC) and gas chromatography-mass spectrometry (GC-MS) from the vanilla root tips extracts. The results showed that higher levels of auxin were found in root tips from old aerial roots and young culture root tips compared to other tissues. The interaction between auxin and cytokinin is important in callus development and differences in the endogenous PGR levels could be the reason for inconsistency in callus responses.

### Cytokinin, The Unorthodox Player in Vanilla Root Callusing

Past research studies on vanilla root culture were scarce. Philip and Nainar (1986) reported the development of direct shoot regeneration from the root tips of vanilla. However, the research paper had focused only on development, but not the practical procedure of micropropagation (Arditti 2009). So far, callus induction in root tips has not been reported. Previous studies on *in vitro* micropropagation of *V. planifolia* had focused on the use of high auxins, while our findings have shown that cytokinin, applied solely and/or in combination with lower auxin ratio, could efficiently induce calli in vanilla root tips. The current study showed that formation of root tips-derived calluses initiated shortly after 90 days, which was about the same period as the callusing rate of vanilla nodes, but at a much higher rate of callusing. Our study therefore shows that root tip could be a potential explant to be utilised when callus regeneration pathway is required.

## CONCLUSION

Our study reported a novel approach to efficiently induce indirect regeneration in vanilla root tips, where up to 100% callusing rate was reported using cytokinin-containing medium. The callus response rate was also higher than nodal cultures, with callus initiation observed within two weeks to one month, as compared to three months in nodal explants by previous studies. This study highlighted the remarkable level of pluripotency of *V. planifolia* root tips and the important role of cytokinin as sole PGR to regenerate plantlet of *V. planifolia* from the root tip through callus and PLB regeneration. The finding will serve as a potential tool for the mass propagation of *V. planifolia* to be utilised both commercially and for further research.

## Figures and Tables

**Table 1:** Effects of different percent and duration of carbendazim for surface sterilisation.

Carbendazim percentage	Bacterial contamination (%)	Fungal contamination (%)	Survival (%)	Phytotoxicity (%)
0.2%	90.00 ± 11.55	50.00 ± 8.16	0.00 ± 0.00	0.00 ± 0.00
0.4%	90.00 ± 8.16	0.00 ± 0.00	10.00 ± 8.16	0.00 ± 0.00
0.6%	90.00 ± 11.55	0.00 ± 0.00	0.00 ± 0.00	10.00 ± 8.16
0.8%	80.00 ± 16.33	0.00 ± 0.00	10.00 ± 8.16	10.00 ± 8.16
1.0%	90.00 ± 11.55	0.00 ± 0.00	0.00 ± 0.00	20.00 ± 14.14

Carbendazim duration	Bacterial contamination (%)	Fungal contamination (%)	Survival (%)	Phytotoxicity (%)

20 min	90 ± 8.16	0.00 ± 0.00	10.00 ± 8.16	0.00 ± 0.00
60 min	10 ± 8.16	0.00 ± 0.00	90.00 ± 14.14	0.00 ± 0.00
120 min	20 ± 16.33	0.00 ± 0.00	80.00 ± 8.16	0.00 ± 0.00
Overnight (16–18h)	20 ± 14.14	0.00 ± 0.00	80.00 ± 16.33	0.00 ± 0.00

*Notes*: Values expressed as means with standard deviations. Each mean was based on 4 replicates, each with consists of 4 cultures.

**Table 2:** Effects of BAP, TDZ, NAA or 2,4-D hormonal treatments on nodal explants when cultured on semi-solid MS medium, after 90 days of culture.

PGR(s)	Concentration (mg/L)	Total response	% callus response	Average shoot number	Type of shoot regeneration
No PGR (Control)		100.00^a^	0.00	1.0^a^	Direct
BAP only	0.5	100.00^a^	0.00^a^	6.0^d^	Direct
	1	100.00^a^	0.00^a^	8.0^f^	Direct
	2.5	100.00^a^	0.00^a^	2.0^b^	Direct
	5	100.00	0.00^a^	0.0^a^	Direct
BAP + NAA	0.5 + 0.5	83.33	0.00^a^	4.0^cd^	Direct
	0.5 + 1.0	83.33	0.00^a^	3.5^c^	Direct
	0.5 + 2.5	83.33	50.0^c^	9.0^fg^	Indirect
	1.0 + 0.5	50.00	0.00^a^	1.0^a^	Direct
	1.0 + 1.0	66.67	0.00^a^	1.5^ab^	Direct
	1.0 + 2.5	66.67	66.67^c^	10.5^hi^	Indirect
	2.5 + 0.5	66.67	0.00^a^	9.8^gh^	Direct
	2.5 + 1.0	16.67	50.00^c^	7.0^ef^	Indirect
	2.5 + 2.5	50.00	50.00^c^	3.5^c^	Indirect
BAP + 2,4-D	0.5 + 0.5	66.67	0.00^a^	1.0^a^	Direct
	0.5 + 1.0	0.00	0.00^a^	0.0^a^	Direct
	0.5 + 2.5	0.00	0.00^a^	0.0^a^	Direct
	1.0 + 0.5	16.67	50.00^c^	7.0^ef^	Indirect
	1.0 + 1.0	16.67	50.00^c^	8.5^fg^	Indirect
	1.0 + 2.5	0.00	16.67^ab^	1.5^ab^	Indirect
	2.5 + 0.5	16.67	0.00^a^	1.0^a^	Direct
	2.5 + 1.0	0.00	0.00^a^	0.0	Direct
	2.5 + 2.5	16.67	0.00^a^	1.0^a^	Direct
BAP + TDZ	0.5 + 0.25	50.00	41.67^bc^	11.5^i^	Indirect
	0.5 + 0.5	83.33	50.00^c^	9.7^gh^	Indirect
	0.5 + 1.0	100.00	50.00^c^	5.6^de^	Indirect
	0.5 + 2.5	83.33	0.00^a^	0.0^a^	Direct
	1.0 + 0.25	100.00	0.00^a^	9.5^g^	Direct
	1.0 + 0.5	66.67	25.00^b^	8.5^fg^	Indirect
	1.0 + 1.0	50.00	25.00^b^	6.0^d^	Indirect
	1.0 + 2.5	100.00	0.00^a^	2.5^bc^	Direct
	2.5 + 0.25	83.33	0.00^a^	1.0^a^	Direct
	2.5 + 0.5	83.33	0.00^a^	1.0^a^	Direct
	2.5 + 1.0	70.00	25.00^b^	3.0^bc^	Indirect
	2.5 + 2.5	60.00	0.00^a^	1.5^ab^	Direct

*Notes*: Values expressed as means of 3 replicates; each replicate consisted of 5 cultures. Different letters within column (lowercase) indicate a significant difference (*p* ≤ 0.05) according to Duncan’s multiple range.

**Table 3:** The influence of sole applications of BAP, TDZ, NAA or 2,4-D hormonal treatments on callus response of root tips explants when cultured on semi-solid MS medium, after 90 days of culture.

Plant growth regulators (PGRs)		Callus response	

PGR type(s)	Concentration (mg/L)	Total response (%)	Average size (mm^2^)
No PGR (Control)		0.0^a^	-
BAP only	0.5	100.00^c^	21.83^a^
	1.0	93.33^bc^	20.50^a^
	2.0	100.00^c^	12.03^a^
TDZ only	0.1	100.00^c^	18.17^a^
	0.25	100.00^c^	18.47^a^
	0.5	90.00^b^	12.67^a^
	1.0	86.67^b^	14.73^a^
NAA only	0.5	0.00^a^	-
	1.0	0.00^a^	-
	2.0	0.00^a^	-
2,4-D only	0.1	0.00^a^	-
	0.5	0.00^a^	-
	1.0	0.00^a^	-
	2.0	0.00^a^	-

*Notes*: Values expressed as means of 3 replicated experiments; each replicate consisted of 10 cultures. Different letters within column (lowercase) indicate a significant difference( *p* ≤ 0.05) according to Duncan’s multiple range test.

**Table 4:** The influence of BAP or TDZ in combination with 2,4-D and NAA hormonal treatments on callus response of root tips explant after 90 days of culture.

PGRs concentration (mg/L)			Callus response	

BAP	NAA	2,4-D	Total response (%)	Average size (mm^2^)
0.5		-	100.00^h^	21.83^fg^
	0.5		90.00^fgh^	15.33^def^
	1.0	-	76.67^efgh^	5.47^ab^
	2.0		26.67^abc^	3.57^ab^
		0.1	0.00^a^	-
	-	1.0	0.00^a^	-
		2.0	0.00^a^	-
1.0		-	93.33^gh^	20.50^fg^
	0.5		76.67^efgh^	5.40^ab^
	1.0	-	83.33^efgh^	19.27^fg^
	2.0		50.00^bcde^	8.30^abc^
	-	0.1	56.67^cdef^	19.57^fg^
		1.0	66.67^defgh^	2.77^a^
		2.0	36.67^bcd^	4.83^ab^

TDZ	NAA	2,4-D	Total response (%)	Average size (mm^2^)

0.25		-	100.00^h^	18.47^efg^
	0.5		100.00^h^	16.50^ef^
	1.0	-	76.67^efgh^	23.87^g^
	2.0		26.67^abc^	15.60^def^
		0.1	76.67^efgh^	17.47^efg^
		1.0	36.67^bcd^	2.87^a^
		2.0	0.00^a^	-
0.5		-	90.00^fgh^	12.67^cde^
	0.5		83.33^efgh^	12.30^cde^
	1.0		50.00^bcde^	5.60^abc^
	2.0		83.33^efgh^	10.10^bcd^
		0.1	60.00^cdefg^	4.73^ab^
	-	1.0	20.00^ab^	8.60^abc^
		2.0	76.67^efgh^	2.70^a^

*Notes*: Values expressed as means of 3 replicated experiments; each replicate consisted of 10 cultures. Different letters within column (lowercase) indicate a significant difference (*p* ≤ 0.05) according to Duncan’s multiple range test
